# Release of Cytochrome C from Bax Pores at the Mitochondrial Membrane

**DOI:** 10.1038/s41598-017-02825-7

**Published:** 2017-06-01

**Authors:** Mingzhen Zhang, Jie Zheng, Ruth Nussinov, Buyong Ma

**Affiliations:** 1 Department of Chemical & Biomolecular Engineering, the University of Akron, Akron, Ohio 44325 USA; 2 Basic Science Program, Leidos Biomedical Research, Inc. Cancer and Inflammation Program, National Cancer Institute, Frederick, MD 21702 USA; 3 Sackler Inst. of Molecular Medicine, Department of Human Genetics and Molecular Medicine, Sackler School of Medicine, Tel Aviv University, Tel Aviv, 69978 Israel

## Abstract

How cytochrome C is released from the mitochondria to the cytosol via Bax oligomeric pores, a process which is required for apoptosis, is still a mystery. Based on experimentally measured residue-residue distances, we recently solved the first atomic model for Bax oligomeric pores at the membranes using computational approaches. Here, we investigate the mechanism at the microsecond time- and nanometer space- scale using MD simulations. Our free energy landscape depicts a low barrier for the permeation of cytochrome C into the Bax C-terminal mouth, with the pathway proceeding to the inner cavity and exiting via the N-terminal mouth. Release is guided by organized charged/hydrophilic surfaces. The hydrophilicity and negative charge of the pore surface gradually increase along the release pathway from the pore entry to the exit opening. Rather than inert passing of the cytochrome C through a rigid pore, the flexible pore may selectively aid the cytochrome C passage. Once the Bax pore is formed in the membrane, with a low energy barrier, the release of cytochrome C may be readily achieved through energy fluctuations. Collectively, our work provides mechanistic insight in atomic detail into the release of cytochrome C through Bax oligomeric pores.

## Introduction

Bax, a pro-apoptotic protein regulator, belongs to the Bcl-2 protein family^1^. It is involved in a wide variety of cellular activities including apoptosis and cancer^1, 2^. In healthy cells, inactive Bax exists in the monomeric state in the cytosol and occasionally at the mitochondrial membranes^3, 4^. Apoptotic signals and external stimulations from abiotic factors including temperature variations, hydrogen peroxide, and pH perturbations, induce Bax’s structural shifts from monomer to dimer, forming heterodimers with the anti-apoptotic Bcl-2 proteins or homodimers^5–11^. Growing evidence suggests that active Bax ensures cell death via pore formation at the mitochondrial outer membranes, giving rise to the release of Cytochrome C to the cytosol, and inducing a series of functional disorders^12–15^.

Cytochrome C is a highly conserved hemeprotein in plants, animals and organisms^16, 17^. Under normal conditions, cytochrome C resides in the inner membrane of the mitochondria, serving as the main component of the electron transport chain^18, 19^. It is also involved in other biological processes, e.g. peroxidase activity, nitrite reduction, and catalysis of hydroxylation and aromatic oxidation^20–24^. Release of cytochrome C from the mitochondria to the cytosol is the key event initiating the apoptotic cascade^16^. After detaching from the mitochondria, cytochrome C binds the IP3 receptors at the endoplasmic reticulum, resulting in local elevation of calcium concentration which facilitates further cytochrome C leakage^25, 26^. When cytochrome C in the cytosol reaches cytotoxic levels, it activates cysteine proteases (caspase 9, caspase 3, caspase 7) and eventually kills the cells^27, 28^.

Formation of Bax oligomeric pores at the mitochondrial outer membranes is prerequisite for cytochrome C release^29–31^ and efforts have focused on the structural morphologies and dynamic properties of Bax membrane pores^32–38^. Activated by the BH3 only protein (tBid or Bim), Bax monomers first attach to membranes, where they oligomerize and gradually insert into the interior of the bilayers^35, 39–41^. Two different scenarios were proposed for Bax insertion, the hairpin model and the in-plane model. These reflect the differences in the orientations of α5 and α6^42^. However, evidence consistently suggested that irrespective of the penetration pathway, Bax eventually forms homodimer pores at the membranes, with the varied pore sizes depending on the concentrations^43^. Using double electron-electron resonance (DEER) spectroscopy in liposomes and isolated mitochondria, Bleicken *et al*. measured residue-residue distances in Bax oligomeric pores at the membranes. They observed the core/Latch domain and proposed helix arrangements in the oligomeric pores^44^.

Based on the residue-residue distances detected experimentally for Bax and its homologous protein (Bak)^45^, we computationally solved the first atomic model for Bax oligomeric pores at the membranes using molecular dynamics simulations^46^. In our model, Bax dimers with dimeric BH3-in-groove conformation associate to form oligomeric pores with the α3:α3′ and α5:α5′ dimer-dimer interfaces, reproducing the experimental residue-residue distances^44, 45^ and opening the way for mechanistic investigation of cytochrome C release at atomic resolution.

While there are multiple pathways for cytochrome C release from the mitochondria in apoptosis^47^, release of cytochrome C through Bax oligomeric pores is the key step^48–50^. Bax induces cytochrome C release by multiple mechanisms in the mitochondria^51^, and modulation of this process is one of the most promising strategies in drug design^52–56^. It has been shown that an antibody binding to the Bak/Bax α1-α2 loop can activate mitochondrial Bax, but blocks translocation of cytosolic Bax^57^. Discovery of small molecules or peptides that directly and selectively regulate these proteins could be a superior method for cytochrome C release^58^. Topologically, Bax assembles into large ring and arcs-like structures^59, 60^. Since activated Bak/Bax can form pores with different sizes^43, 60^, the question arises whether the passage of cytochrome C through the pore a generic passive process or is cytochrome C-selective, and if selective, how. Atomic insight into the release process of cytochrome C from Bax oligomeric pores at the membranes is important, because not only this process is pathological crucial, but also the information may provide the structural and dynamic properties that may benefit the Bax- and cytochrome C-targeting drug design. However, the experimental characterization of this process is infeasible due to both the complexity of the membrane environment and the transient nature of the release process. Conventional MD simulation cannot be applied to probe this release process, because of the insufficient sampling space and efficiency. Using the coarse-grained model and replica-exchange simulation make it possible to probe the whole process at the microsecond time- and nanometer space-scale, allowing depicting the whole permeation process of cytochrome C from Bax pores in the full free energy landscape.

In this work, we computationally investigated the release of cytochrome from Bax oligomeric pores at the membranes, by both coarse grain replica exchange and explicit-solvent all-atom MD simulations. We characterized the free energy pathway of releasing cytochrome C from Bax oligomeric pores, and determined the spatial orientation of Bax oligomeric pores relative to the mitochondria. Our results show that cytochrome C is released from Bax oligomeric pores via three steps: (i) cytochrome C is initially anchored at the wider opening of the oligomeric pores, mainly associating with the C-terminal α6-α8 of Bax; (ii) cytochrome C detaches from the wider opening and approaches the inner pore cavity, tightly binding to α4 of Bax; (iii) cytochrome C penetrates the inner cavity and reaches the narrower pore opening, contacting Bax N-terminal α2-α3 regions. During cytochrome C permeation, the flexibility of Bax oligomeric pore accommodates cytochrome C interaction, morphologically changing but still maintaining the overall structural integrity. Surface and residue-residue contact analysis reveal that cytochrome C permeation is driven by electrostatic forces along the pore.

## Results and Discussion

### Bax hexa-dimer oligomeric pore maintains an integral channel to allow passing of cytochrome C

**Figure 1:**
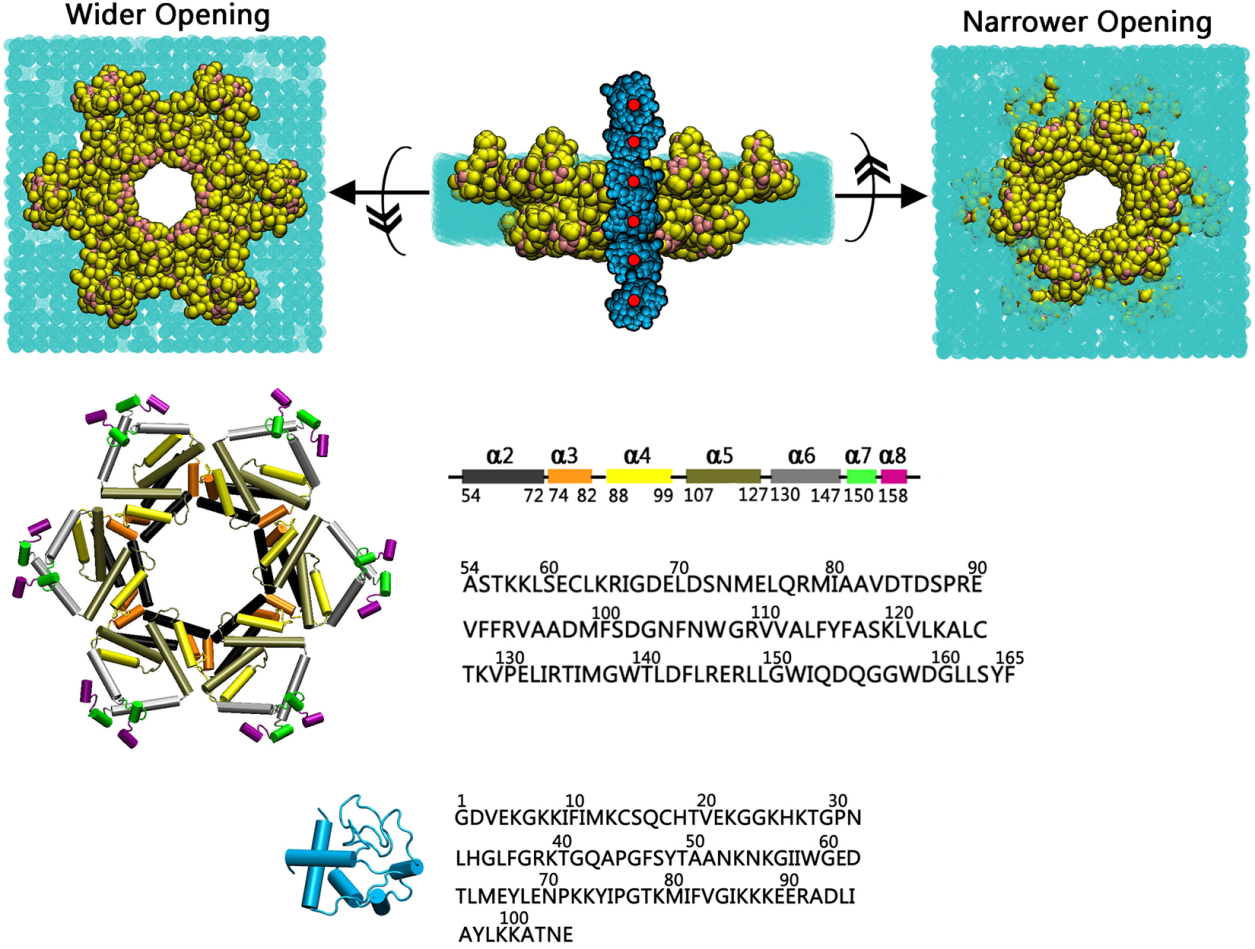
Snapshots of coarse-grained Bax oligomeric pores (yellow and pink) with the cytochrome C (cyan) at the membranes (green) (top); Secondary structures (Cartoon) and the amino acid sequences for Bax and cytochrome C (bottom).

Figure 1 summarizes the simulated systems and the modeling protocols. In this study, we selected a truncated Bax oligomeric pore lacking both N-terminal α1 and C-terminal α9 segments. Previous studies have shown that C-terminal (α9) truncated Bax has channel-forming activity at the liposomes, inducing the release of cytochrome C similar to the native full-length Bax^41^. Evidence also suggested that N-terminal (α1) truncated Bax preserves its apoptotic capacity^61^, showing higher cell toxicity than the native Bax^62^. These results suggested that the terminal truncated Bax can form typical oligomeric pores at the membranes and release cytochrome C from the mitochondria. Structurally, α9 resides at the outer surface of the pores, entirely embedded in the lipid bilayers^46^. It may barely contact with the released cytochrome C, thus insignificantly influencing this process. The positions and orientations of α1 regions are still inconclusive, lacking experimental data. Thus, α1 and α9 are not considered here, eliminating the uncertainty and potential bias in the simulations and meanwhile reducing the computational load. Modeling of the truncated Bax oligomeric pores balances computational efficiency and accuracy. As shown in Fig. 1, Bax oligomeric pore at the membranes exhibits an asymmetrical conformation at the membrane surface plane (x, y) but asymmetrical morphology along with membrane normal (z) with one wider opening covering α6- α8 and another narrower opening covering α2-α4. The Bax six-dimer oligomeric pore has an inner cavity at the center with the diameter around 48 Ǻ, consistent with experimental data^45^. Such a pore-like inner cavity is larger than the size of cytochrome C (~36 Ǻ), thus may well accommodate the incoming cytochrome C and allowing its theoretically free release.

**Figure 2:**
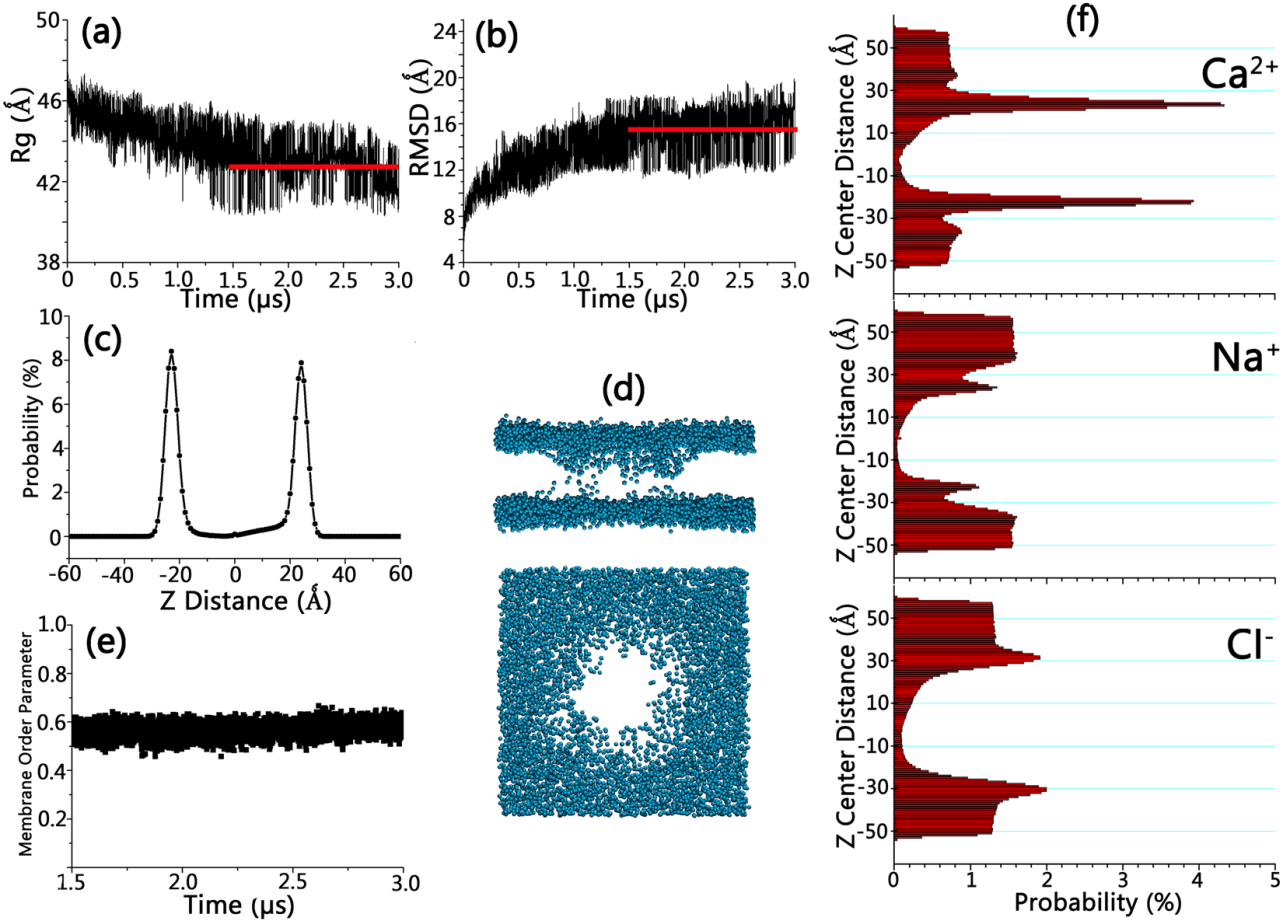
Time-dependent Rg and RMSD profiles for Bax oligomeric pores and cytochrome C (**a**,**b**), distribution probability profile for PO_4_ groups of the lipids (**c**), superimposed snapshots for PO_4_ groups of the lipid bilayers (**d**), membrane order parameter (**e**), and the distribution probability profiles for ions (**f**). Z distance: vertical distance to pore center alone the pore axis.

The replica exchange simulation of Bax pore/cytochrome C was run for 3.0 μs at temperatures ranging from 309 to 380 K, in which the system successfully achieved equilibrium after 1.5 μs, as evidenced by the plateaus of the time-dependent radius of gyration (Rg) and root-mean-square deviation (RMSD) profiles in Fig. 2a,b. The equilibrium trajectory showed that the simulation systems maintained reasonable structural arrangements. The decrease of Rg and increase of RMSD indicated that cytochrome C induced pore shrinkage. However, the structural integrity of the Bax oligomeric pores was well maintained, without any obvious dimer-dimer disassociations. The distribution of lipid PO_4_ headgroups and membrane lipid order parameter were calculated. As shown in Fig. 2c, two predominating peaks exist at ± 24 Ǻ in the PO_4_ headgroup distribution profiles, consistent with a 4.98 nm membrane thickness from the small-angle neuron scattering measurement^63^. While missing in solution (z < −30 Ǻ and z > 30 Ǻ), the PO_4_ groups exhibit certain distribution in the interior space of the membrane. Snapshots of the simulation systems in Fig. 2d confirmed the existence of the PO_4_ groups in the membrane interior and suggested that Bax retains a toroidal pore shape in the membrane, in line with previous experimental observations^32^. Lipid alignments in the bilayers are quantified by the membrane order parameters. Order parameters were calculated by P_x_ = 0.5*(3*cos^2^(τ) −1), which calculates the angle (τ) between the positional vector connecting lipid carbon chain and the bilayer normal^64^. P_x_ reflects the lipid alignment relative to the membrane normal, i.e. P_x_ = 1 means perfectly ordered lipids, while the P_x_ = 0 means randomly mixed lipids. Previous work shows that the membranes without disruptions have the Px ranging from 0.75–0.80^65^. While the toroidal pore formed in the membranes, the lipids adjacent to the Bax pores rearrange their orientations with the headgroups buried in the hydrophobic areas of the membranes. Such rearrangement may disrupt the lipid alignment, reducing Px values. Even though it is not possible to quantitatively correlate the second-rank order parameter with a toroidal configuration, Fig. 2e shows that the lipid order parameters for DSPC membranes consistently fluctuated around 0.60.

The distributions of the ions were also monitored for the equilibrated systems along with the inner cavity of the Bax pore (z axis). NaCl and CaCl_2_ counter ions at 0.035 M were added to mimic a 0.15 M ion concentration. Ion distribution curves (Fig. 2f) show that in solution (z > 30 Ǻ and z < −30 Ǻ), the distributions for all ions are homogenous, but become uneven at the membrane regions (−30 Ǻ < z < 30 Ǻ). At the membrane surface areas, Ca^2+^ showed two protruding distribution peaks which are much higher than those in solution. Visual examination of the simulated systems showed that the majority of the Ca^2+^ ions in the systems strongly associate with and neutralize the anion PO_4_ headgroups in the lipids. While a strong Ca^2+^ layer formed at the membrane surface area, it stabilizes the membrane surface by both forming the PO_4_^−^-Ca^2+^-PO_4_^−^ dual salt bridges and blocking the penetration of other cations. Thus, the Na^+^ at the membrane surface was reduced as compared to solution. The extra charges from Ca^2+^ at the membrane surfaces were compensated by the Cl^−^ ions, as illustrated by its minor peaks at the membrane surface. The preference of Ca^2+^ at the membrane surface implies potential Ca^2+^ roles in modulating the Bax oligomerization at the lipid bilayers, in line with previous experiments^66–68^. Importantly, the distributions of all ions in the inner cavity of the pores (−30 Ǻ < z < 30 Ǻ) point to the importance of ions in neutralizing the charged Bax surfaces and their potential roles in cytochrome C release^26^.

### Permeation Pathway of Cytochrome C through Bax pore

**Figure 3:**
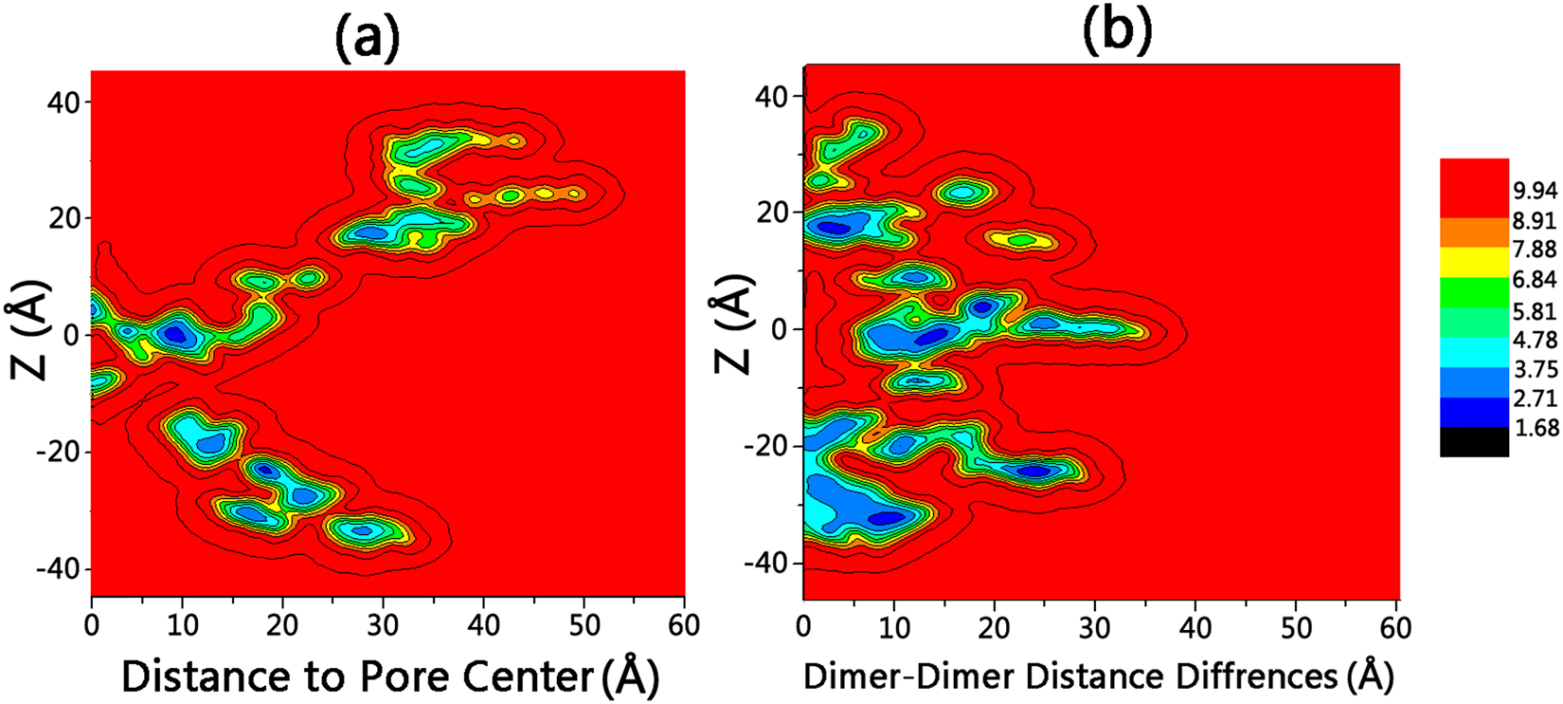
Two-dimensional (2D) free energy landscape for the release of cytochrome C from Bax oligomeric pores at the membranes. Z distance: vertical distance to pore center alone the pore axis. Left panel: X-axis: distance of the center of mass of cytochrome C to the center of pore. Right panel Characterizing of Bax pore elliptical shape by the differences between the longest and shortest distance between the center of masses of two opposite dimers in hexagon vertices. A larger dimer-dimer distance difference refers to a larger shape change of the pore from regular hexagon.

Based on the equilibrium trajectories of REMD simulation of the Bax pore/cytochrome C system, we calculated the two-dimensional (2D) free energy landscape for the release of cytochrome C by -RTlog (P_xy, z_), where P(_xy, z_) is the probability for the cytochrome C at the position with the xy distances at the membrane surface plane and z distance along with the Bax inner cavity relative to the center of Bax pore. The free energy landscape in Fig. 3 indicates that the release of cytochrome C is a multi-step process, involving intermolecular associations between cytochrome C with the entire surface areas of the Bax pores. The lack of the potential basins in the Bax-free regions and the continuous potential local minima at the Bax surfaces suggested that while approaching to the Bax oligomeric pores, cytochrome C tends to firstly attach on the edge surface of Bax pores, then gradually roll in and pass through the inner cavity. Even though the free pore size is larger than the size of cytochrome C, it is unlikely that it goes freely through the Bax pore without contacting the pore openings.

**Figure 4:**
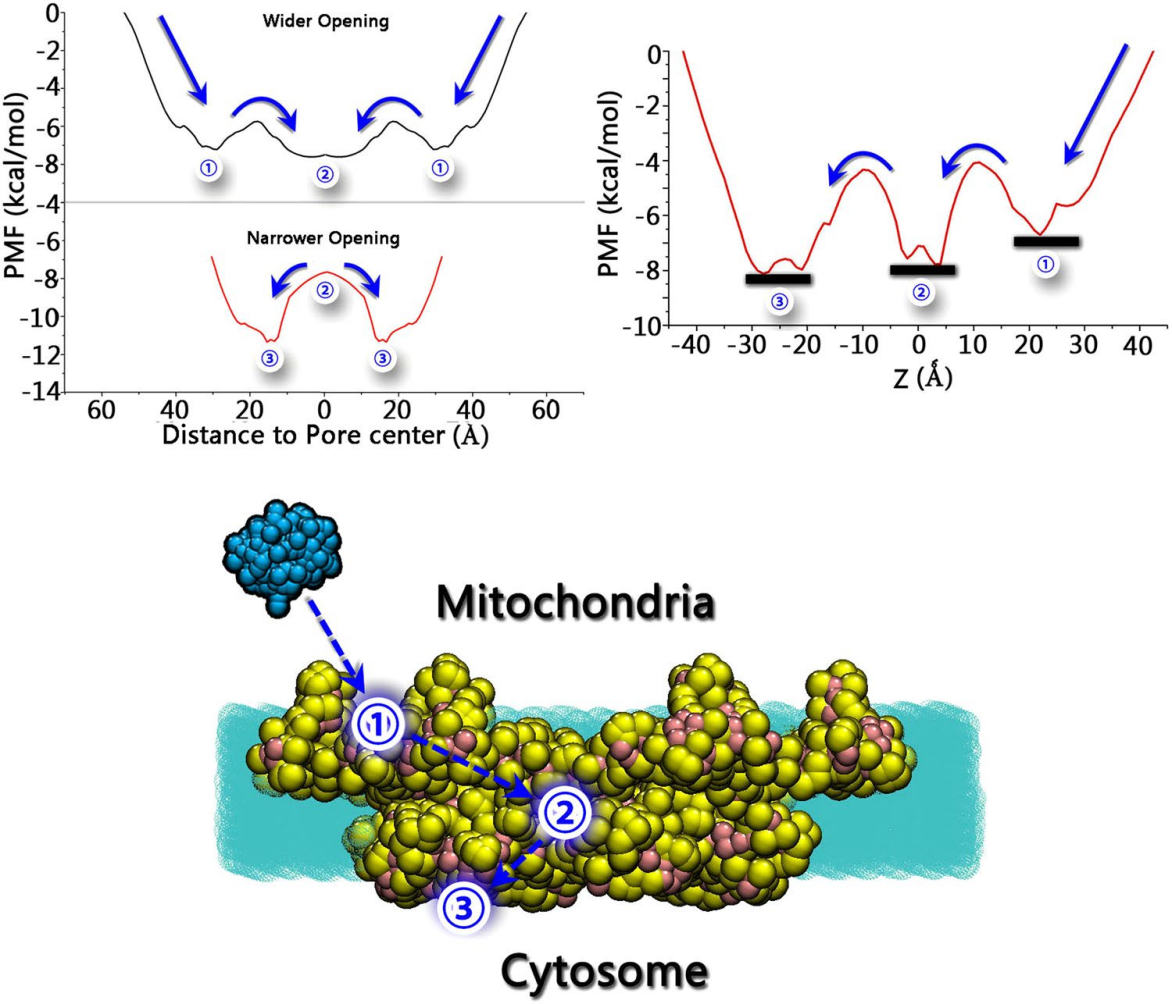
One-dimensional (1D) potential mean force (PMF) profiles along with xy distance and z axis (top), and the schematic diagram illustrating the release pathway of cytochrome C through Bax oligomeric pores. The red plateaus in the Fig. 3 were taken as the energy references (zero energy), since the sampling in red plateaus equals to zero. Thus, the zero energy appeared at z = −40 or 40 and XY = 55. Color code: cytochrome C (cyan), Bax oligomeric pores (yellow and pink), and membranes (green). ① denotes the Bax areas at 25 Ǻ < xy < 40 Ǻ and 15 Ǻ < z < 30 Ǻ. ② denotes the Bax areas at 0 Ǻ < xy < 10 Ǻ and −5 Ǻ < z < 5 Ǻ. ③ denotes the Bax areas at 10 Ǻ < xy < 25 Ǻ and −30 Ǻ < z < −20 Ǻ.

In order to estimate the activation barrier for cytochrome C release, the 2D free energy landscape was further decomposed into one-dimensional (1D) potential-mean-force (PMF) profiles based on the three stages of cytochrome C release: 1. before pore entry; 2. inside the pore; and 3. after leaving the pore (Fig. 4). The PMF profiles revealed that there are three local energy minima corresponding to the three stages of cytochrome C release. The three potential energy basins are located at ① (25 Ǻ < xy < 40 Ǻ, 15 Ǻ < z < 30 Ǻ, at the wider opening of Bax pores), ② (0 Ǻ < xy < 10 Ǻ, −5 Ǻ < z < 5 Ǻ, at the center of Bax inner cavity), and ③ (10 Ǻ < xy < 25 Ǻ, −30 Ǻ < z < −20 Ǻ, at the narrower opening of Bax pores), respectively. As shown in Fig. 4, state ③ is the most energetically favored, followed by ② and ①. The release of cytochrome C via Bax pore depends on the gradients of concentration, free energy minima and other factors, maximizing the system entropy^69^. The maximization of entropy by the concentration gradient between the mitochondrion and cytosol could be an initial driving force for Cytochrome C release. However, the rapid release of the entire Cytochrome C pool from mitochondria still need a favorable free energy guidance. A descending free energy may benefit the cytochrome C release from Bax pores, by providing the asymmetry in the flux. Thus, release of cytochrome C is highly possible to follow the pathway of ① → ② → ③. This suggests that when released, cytochrome C has to first approach the wider opening of the pores from the intermembrane space, reaching the first potential energy basin. Then, it gradually moves to the center of the pores, stabilized by the interaction with Bax residues. Finally, cytochrome C departs from the inner cavity to the narrower opening of the pores, completing the release process. Importantly, the energy barriers between the adjacent potential basins are only around 1 to 4 kcal/mol (Fig. 4), indicating that the release of cytochrome C through the Bax pores may be readily achieved under normal energy fluctuations in the systems.

Bax oligomeric pore has an asymmetrical geometry along the membrane normal. Previously, the orientation of the pores and its biological implication were elusive. The proposed pathway suggests that the orientation in the mitochondrial membranes determines the release mechanism of cytochrome C. Our proposed pathway from simulations shows that the wider opening of Bax pores located at the intermembrane space of the mitochondria is responsible for the initial anchoring of cytochrome C, and the narrower opening exposed to the cytosol may be exiting path for its release.

### Key residues stabilize the intermediates during cytochrome C release

**Figure 5:**
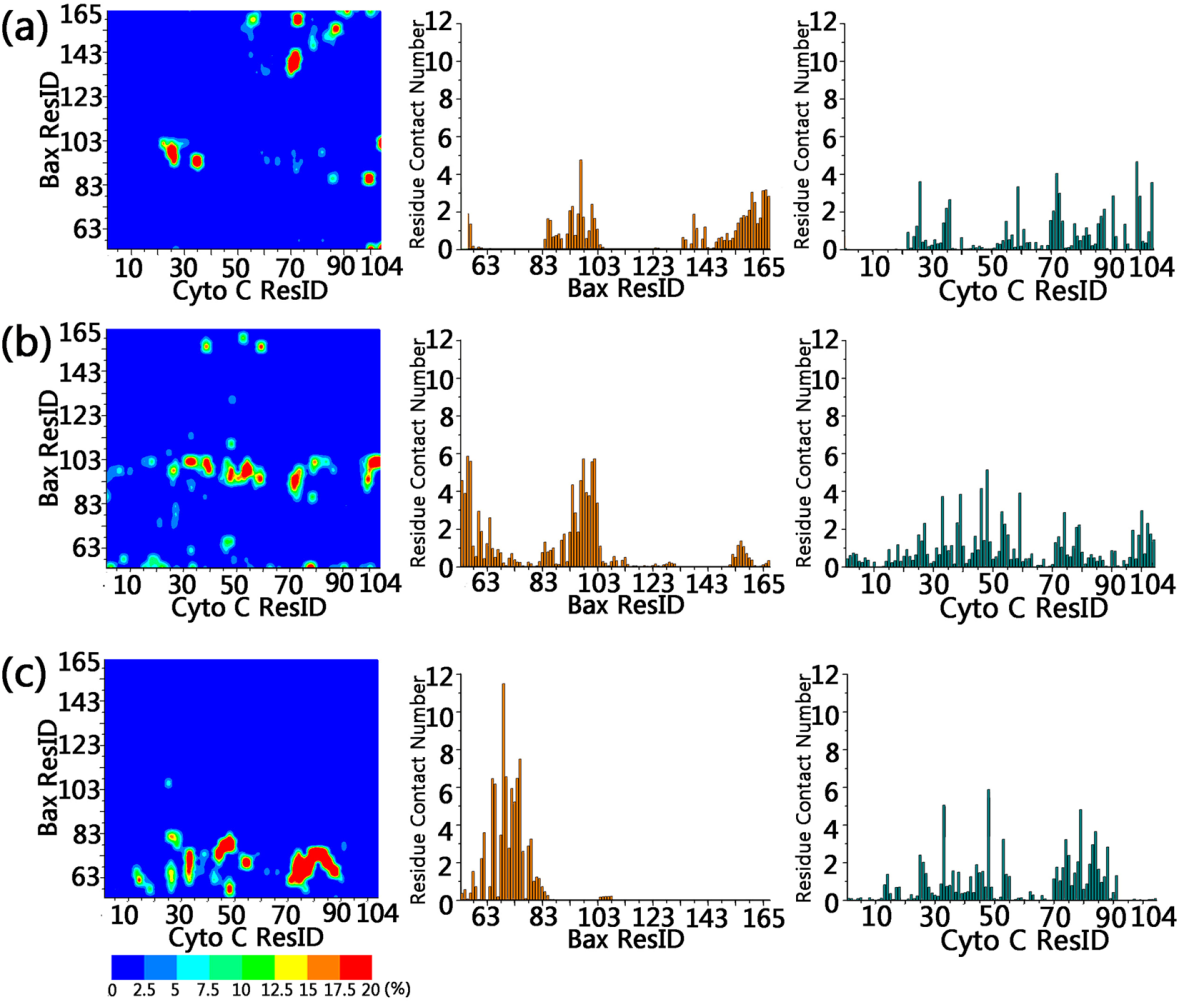
Residue-residue contacting matrix (left), and the residue contact number profiles for Bax (middle) and cytochrome C (right) at ① (**a**), ② (**b**) and ③ (**c**).

The association between cytochrome C and Bax oligomeric pores may be governed by interfacial residues of both partners along the permeation stages. To identify the key residues and examine their significance, we calculated the residue-residue contacting matrix with residue-residue distance <6.5 Ǻ. Figure 5a,b and c show the 2D residue-contacting maps for ①, ② and ③, respectively, followed by contacting probability plots for individual pore and cytochrome C residues.

REMD trajectories showed that at the wider opening of the pore (①), interfacial residue binding mainly occurred at two regions, between the C-terminal amino acids of the pores (residue 130–165, α6-α8) and cytochrome C (residue 50–90), and between residue 83–105 (α4) of Bax oligomeric pores and residue 23–38 of cytochrome C, whereas other parts of both partners (N-terminal residues of the Bax pore and cytochrome C) are rarely involved. These simulation results suggest that the α4, α6-α8 of Bax pores are important for the initial anchoring of cytochrome C. Moving to the inner cavity of the pore (②), cytochrome C has close contact with the α4 (residue 83–105). Stage 1 contacts with α6-α8 of the pores were replaced by the binding between the N-terminal residues 54–75 (α2) of Bax and cytochrome C, suggesting that the translocation from ① to ② involves the separation of cytochrome C from α6-α8 to the α2 helix of Bax. Approaching the exit opening (③), the binding residues of the pore further shifted to the N-termini. The binding between cytochrome C and α4 disappeared. Instead, cytochrome C tends to solely bind to residues 54–85 (α2–α3) of Bax. The binding residues in cytochrome C also exhibit changes. Different from ① to ②, both N- and C-terminal residues in cytochrome C become free from Bax associations, implying a new binding model at this region.

### Structural adjustments and driving forces for cytochrome C permeation

**Figure 6:**
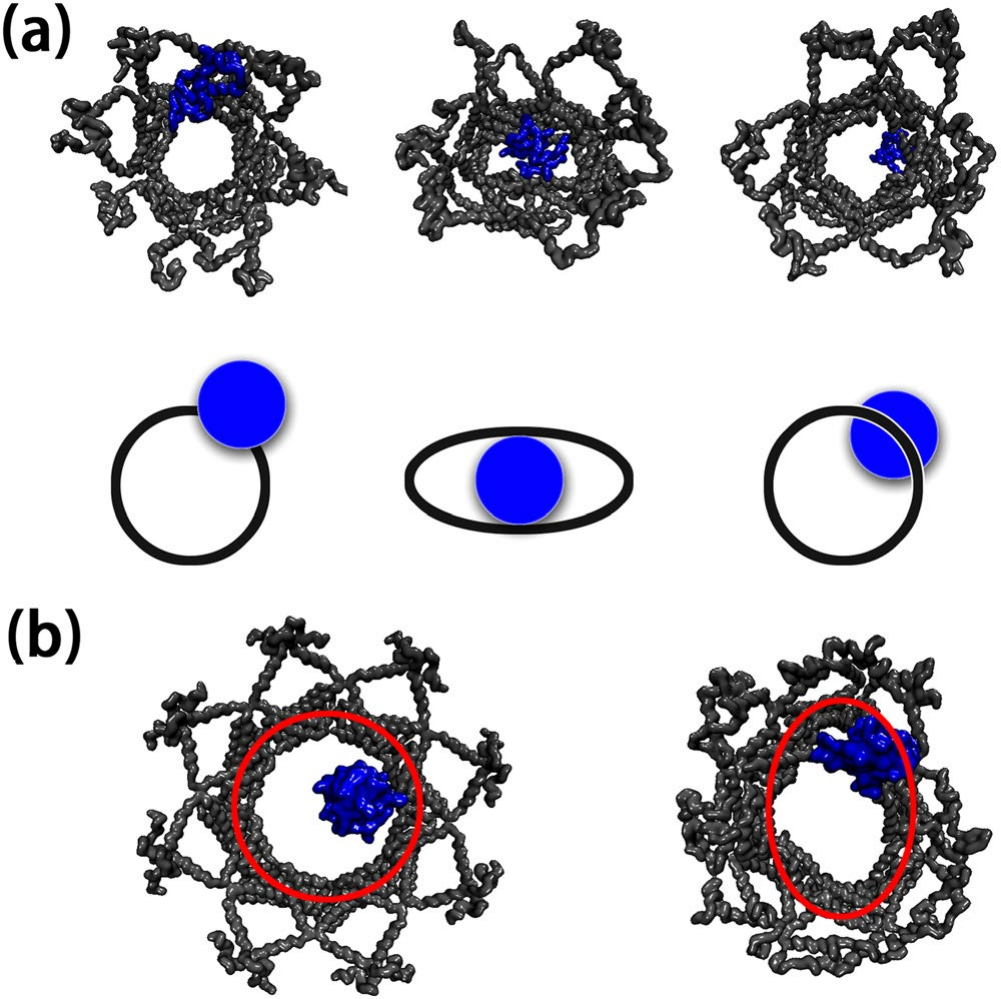
Snapshots for the simulated six-dimer Bax pores at ① (top left), ② (top middle) and ③ (top right), and the schematic diagram highlighting the morphological changes of Bax oligomeric pores upon binding to cytochrome C (bottom), and (**b**) the initial and final structures of eight-dimer Bax pores. Color code: Bax oligomeric pores (black), cytochrome C (blue).

Previous experiments suggested that cytochrome C may induce pore morphological changes during its release process^11, 30^. The exposure to cytochrome C at the mitochondrial membrane may increase the diameter of the pores^30, 33^, suggesting considerable pore flexibility to fit and accommodate incoming cytochrome C molecules. A similar phenomenon was observed in our simulations. Figure 6a shows typical snapshots at ①, ② and ③, respectively. As shown, while cytochrome C resided at both wider and narrower openings, the pore maintained its initial morphology with a constant circular inner cavity. However, as cytochrome C approached the center of the inner cavity, it changes from round to oval shape. To check change of shape as a variable in energy landscape accompanying cytochrome C release, we characterize Bax pore elliptical shape by the differences between the longest and shortest distance between the center of masses of two opposite dimers in hexagon vertices. A larger dimer-dimer distance difference refers to a larger shape change of the pore from regular hexagon. The 2D free energy landscape taking the shape change and Z distance as the reaction coordinates is shown in the right panel of Fig. 3. The results clearly showed that dimer-dimer distance differences are much larger while cytochrome C is in the middle of the Bax pores, compared to those with cytochrome C at both entrancing and exiting mouths of the Bax pores. Theoretically, a small inert protein can pass through the Bax pore unselectively. However, we observed specific interactions between Bax and cytochrome C, forming a stable intermediate and triggering pore reshaping. Despite the conformational changes induced by cytochrome C at the cavity center, the pore preserved its structural integrity. The dimer-dimer associations in the pores were well kept, without obvious disruptions. This implies that release of cytochrome C is indeed capable of inducing the morphological change of Bax oligomeric pores, in consistent with experiments^30, 31^.

**Figure 7:**
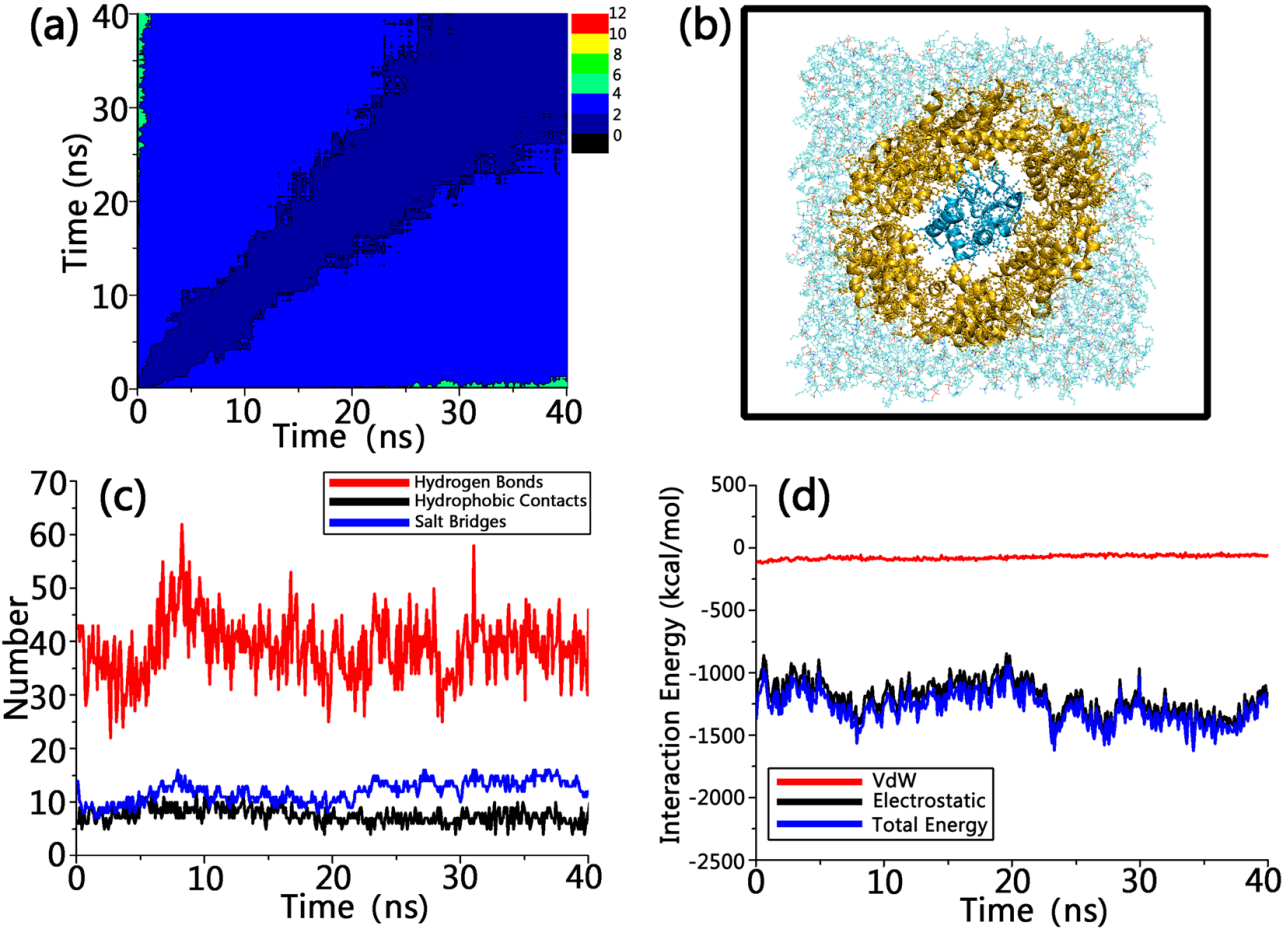
2D RMSD matrix (**a**), snapshots (**b**), non-bonded analysis (**c**) and interaction energies (**d**) for the all-atom explicit-solvent simulations of the cytochrome C in the inner cavity of Bax oligomeric pores.

To further study the cytochrome C-induced structural changes, using the REMD equilibrated structure in the Martini model, we reconstructed an all atom cytochrome C/Bax pore structure and re-equilibrated the structure using all-atom MD simulations. The simulated all atom structure presents excellent structural stability throughout the 40 ns simulations. The 2D RMSD plots in Fig. 7a show that both pore and cytochrome C experienced minor structural changes, with the RMSD values consistently lower than 4 Ǻ. At 25 ns, the systems achieved equilibrium. After that the proteins become more steady, as evidenced by the much lower RMSD values <2 Ǻ. Figure 7b shows the final snapshots for the simulated systems. It can be seen that the helix-abundant conformations and the structural integrity for both the pore and cytochrome C were maintained, presenting compact protein-protein interactions between neighboring Bax dimers and between the Bax pore and cytochrome C, confirming the results from coarse grain REMD simulations. ~25% surfaces of cytochrome C was embedded by the inner cavity. Non-bonded analysis (Fig. 7c) showed that the hydrogen bonds, salt bridges and hydrophobic contacts synergistically enhanced the interfacial binding between cytochrome C and Bax oligomeric pore. In comparison, hydrogen bonds (~40) and salt bridge (~13) have larger contributions than the hydrophobic contacts (~8), suggesting that selective inclusion of cytochrome C into the inner cavity of the pore is controlled by electrostatic interactions. As shown in Fig. 7d, ~95% of the total interaction energies derived from the electrostatic forces, while the VdW forces only contributed ~−100 kcal/mol.

Since the sizes of the Bax oligomeric pores may vary, the Bax can form higher oligomer assemblies of 50–60 angstrom pores to release cytochrome C^70^. We also conducted the simulation of the larger Bax pores with eight dimer motifs at the membranes, and simulated it in presence of cytochrome C. As shown in Fig. 6b, the similar structural changes for Bax oligomeric pores with initial pore size of ~61 Ǻ were observed, suggesting that the active structural changes may be a general phenomenon for Bax pores to release the cytochrome C regardless of their sizes.

**Figure 8:**
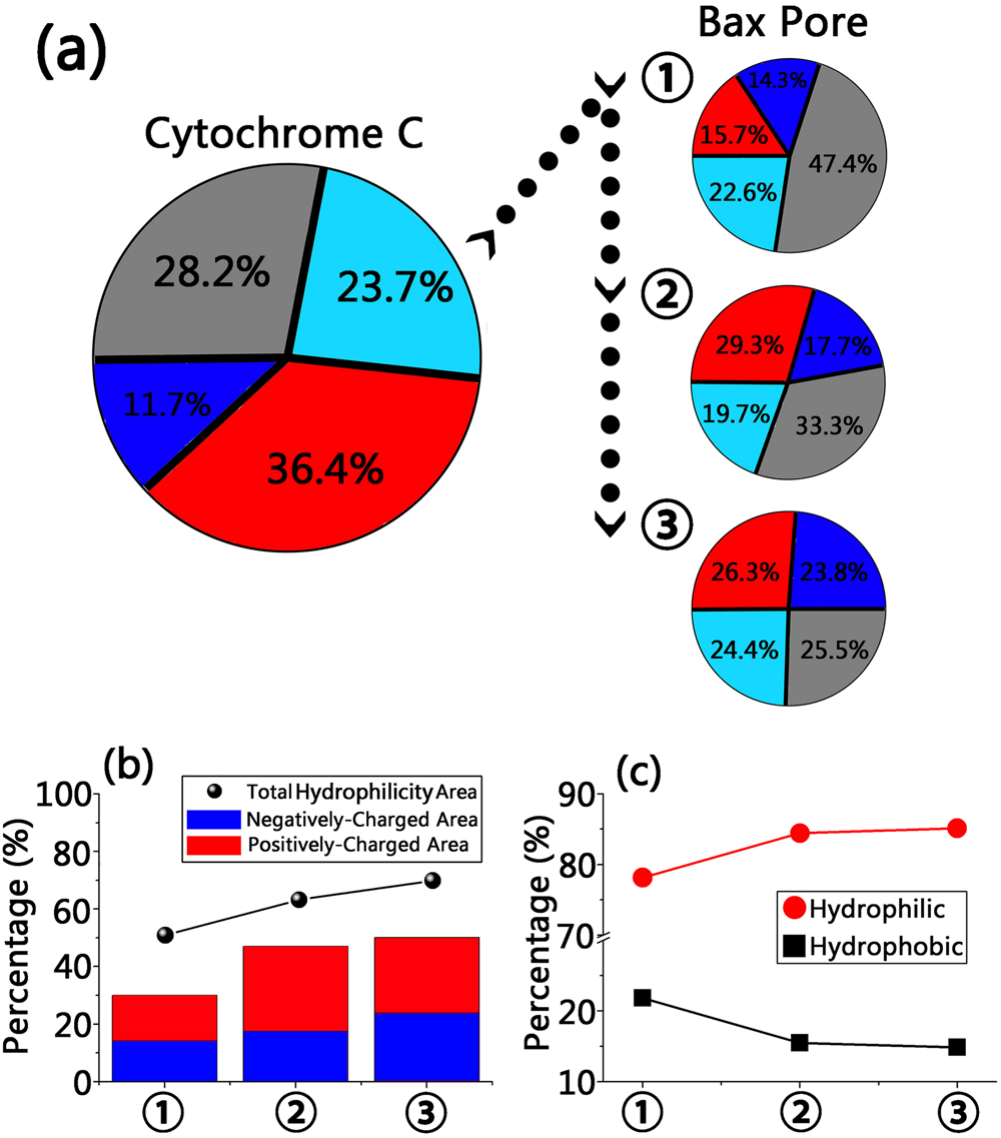
Surface properties for the cytochrome C and Bax oligomeric pores at different locations (**a**, color code: positively-charged surface (red), negatively-charged surface (blue), non-charged hydrophilic surface (cyan), hydrophobic surface (gray)), sum of hydrophilic areas (**b**), and the percentages of hydrophilic-hydrophilic and hydrophobic-hydrophobic residue-residue contacts between cytochrome C and Bax oligomeric pores at different locations (**c**).

The external surfaces for both cytochrome C and the Bax oligomeric pore are the primary and major interacting sites determining the release landscape. As shown in Fig. 8a, cytochrome C, as a natively folded protein, consists of 72.8% hydrophilic surfaces and 28.2% hydrophobic area. Further decomposition of the hydrophilic surfaces showed that the positively-charged (36.4%) surfaces occupied much higher percentage than the negatively-charged (11.7%), indicating that cytochrome C has positively-charged hydrophilic surfaces. Similarly, the solvent-exposed surfaces of the Bax pore are hydrophilic and charged, but to different extent at different locations (Fig. 8a,b). At the wider opening sides (①), the hydrophilic areas occupied 52.6% of total solvent-accessible surfaces, with 15.7% positively-charged, 14.3% negatively-charged and 22.6% non-charged areas. The inner cavity of the pores (②) has larger hydrophilic areas of 66.7%, contributed by the increased positively-charged (29.3%) and negatively-charged (17.7%) areas. This becomes more pronounced for the narrower opening of the pore (③), whose hydrophilic surfaces increased to 74.5% and has the largest negatively-charged surfaces (23.8%). Thus we observed an increasing gradient of negatively-charged patch from the entry opening through the pore center to the exit opening.

Figure 8c presents the percentages of hydrophilic-hydrophilic and hydrophobic-hydrophobic residue-residue contacts at ①, ② and ③. At any region of the pore, the hydrophilic-hydrophilic residue contacts have dominant percentages, 4–6 times higher than hydrophobic-hydrophobic residue interactions. Along the release pathway from ① to ② to ③, the percentage of hydrophilic-hydrophilic contacts continuously increased from 77.2% to 83.3% to 85.2%, suggesting that enhanced polar residue contacts may drive the cytochrome C through the pores. Therefore, the increase of hydrophilic surfaces along with the release pathway, combined with the hydrophilic nature of cytochrome C, reveal that electrostatic forces between the polar residues are the main driving force for the release of cytochrome C.

## Discussion

The release of cytochrome C through Bax oligomeric pores at mitochondrial membranes mediates apoptosis and serves as a target for the drug discovery against diseases, including cancers^2^. Despite decades of investigation, the activation, regulation and function of Bak/Bax during apoptosis are still unclear, as is what are the mechanism and detailed pathways of cytochrome C passing through Bak/Bax pore. Experimental investigation at atomic resolution is still infeasible. Based on replica exchange molecular dynamics simulations, we examined the overall free energy landscape for the release of cytochrome C through the Bax oligomeric pores. Our results suggest that the release of cytochrome C follows the pathway of wider opening → inner cavity → narrower opening through Bax oligomeric pores. Bauer and Nadler have demonstrated that binding sites in a channel can facilitate transport with a simple mechanism that once the single molecule is bound by the binding site, its escape is faster to the closest side^69^. Consistently, we found that during its releasethis process, cytochrome C gradually binds to the C-terminal α6-α8, central α4, and N-terminal α2-α3 segments of Bax. Our analysis of the surface properties of cytochrome C/Bax oligomeric pore indicated that electrostatic match could control and drive selective release of the positively-charged cytochrome C. The Bax pore has an organized distribution of charged hydrophilic surfaces at different locations. The hydrophilicity and negative charge of Bax pore surfaces continuously increases along with the release pathway of cytochrome C from the pore entry to the exit opening, facilitating the selection and permeation of cytochrome C. We observed that the incoming cytochrome C induces structural adjustment of the Bax oligomeric pore, arguing that it does not pass inertly through a rigid pore. The induced fit between cytochrome C and the Bax oligomeric pore explains that the varied pore sizes may point toward specificity in cytochrome C release. Consistent with fast cytochrome C release^71^, the energy barrier through the pathway is less than 4 kcal/mol. Thus, once Bax pore is formed in the membrane, the release of cytochrome C through it may be readily achieved via normal energy fluctuations.

Considering the complexity of the release of cytochrome C, there are several important factors that could potentially modify the theoretical mechanism. In our simulations, we used coarse grained Martini force field combined with all atom CHARMM36 force field. The applications of Martini force field have successfully generated insightful and reliable protein dynamics and free energy surfaces^72^, including in membrane environments^73, 74^.

The Bax pore sizes depending on the concentrations^43^, and we modeled a hexamer of dimer. In selection of simulation system, we also examined a pentamer of dimer, which has a pore diameter with similar size of cytochrome C. Our selection of hexamer of dimer allow us to examine the possibility of both free and selective passing. For the pore with larger size, the surface electrostatic interactions with cytochrome C should still operate and the interaction still could trigger adjustment of pore size to selectively allow cytochrome C permeation. Indeed, our room temperature simulation of the larger Bax pores with eight dimer motifs at the membranes in presence of cytochrome C confirmed the shape change of Bax pore. The flexibility of pore is the results of the relative motion between different Bax proteins combined with the internal flexibility of each individual Bax, and influenced by cytochrome C interaction. This kind of allosteric communication and regulation mechanism is very common in protein function^75, 76^.

In our modeling of the Bax/Bak pores, we have shown that orientations of the α1 (residues 1–53) and α9 (residues 166–192) segments are in the outmost region of the pore, consistent with the distance measurements observed in experiments^46^. Several experiments have shown that α1 and α9 are not constituents of interior pore, which are mostly central helices^42, 77, 78^. It has been shown that Bax α-helices 2–5/9 is the minimum domain required for oligomerization and apoptotic function^79^, and that the core α 2–5 helices of Bak are sufficient for dimerization but that the α 6–8 helices are essential for Bak function^77^. Lyer *et al*. also shown that Bak α9 traverses the MOM but does not line a pore following apoptosis^78^. Therefore, the omission of α1 and α9 in our current work does not affect our evaluation of release of cytochrome C through the Bax/Bak pore.

In conclusion, our simulations revealed that the release of cytochrome C from Bax oligomeric pores is an active permeation process with three characteristics. First, we found there is only a low energy barrier of the release of cytochrome C, suggesting a rapid release that may be readily achieved through energy fluctuations. Second, these free energy is funneled towards release of cytochrome C by the delicate match between the hydrophilicity and negative charge of Bax oligimeric pore surfaces and the positively-charged cytochrome C surfaces. These binding sites between Bax pore and cytochrome C provides excellent example of Bauer and Nadler’s theory that binding sites in a channel can facilitate transport. Finally, we found that Bax oligomeric pore can change shape to accommodate interactions between Bax pore and cytochrome C. The morphological change of Bax oligomeric pores explains that the varied pore sizes may point toward specificity in cytochrome C release, which can also be applied to the larger AIF, SMAC and Diablo.

## Materials and Methods

### Bax Oligomeric Pores and Cytochrome C

Bax oligomeric pores were modeled according to the double electron-electron resonance (DEER) residue-residue distances^41, 45^, and the crystal structures of the Bax monomer (1F16)^3^ and BH3-in-groove dimer (4BDU)^5^ as templates. Six Bax homo-dimers associate in the pores via α2:α3 and α4:α5 surfaces, with the α2-α5 BH3-in-groove framework and the anti-parallel model of α6-α8 domain^45^ (Bax oligomeric pore modeling details can be found in our previous work)^46^. For efficiency and accuracy, in this work, we selected the truncated Bax six-dimer oligomeric pore, lacking the α1 (residues 1–53) and α9 (residues 166–192) segments (Fig. 1). The pore was initially modeled using the all-atom model, relaxed by multi-step minimizations and short explicit-solvent simulations using the CHARMM 36 force field. Then, the all-atom coordinates of Bax oligomeric pore were mapped into the coarse-grained model by the Martini force field (version 2.4). Consistent with our previous work^46^, coarse-grained distearoyl-phosphatidylcholine (DSPC) lipids were used to generate the mimicked mitochondrial membranes, since phosphatidylcholine (PC) is their most abundant lipid, occupying over 44% weight of the total phospholipids^80^. Initial all-atom coordinates of cytochrome C were obtained from the crystal structure (3ZCF)^81^, which were mapped into the coarse-grained model using the Martini force field (version 2.4). Coarse-grained water beads were employed to solvate the system with 15 Ǻ distance between the edge of box and the solvent atoms. NaCl and CaCl2 of 0.035 M concentrations were added into the system to mimic the 0.15 M ion strength in solution.

To establish the initial conformations for the replica exchange simulations, the coarse-grained cytochrome C molecules were placed at six different positions along the pore inner cavity (z axis) with a distance interval of 13 Ǻ (Fig. 1). At each point, eight orientations were randomly generated. A total 48 independent replica systems were established. Excluding candidates in the Bax inner cavity, cytochrome C at both wider and narrower openings initially did not contact with the pore surfaces.

### Coarse-grained Replica Exchange Simulation Protocols

Coarse-grained replica exchange simulations were performed using the Gromacs-4.6.5 program with the Martini force field (version 2.4). The independent 48 replica exchange systems were exchanged at temperatures ranging from 309 K to 380 K. The exchange between two replicas was attempted every 1000 integration steps. Based on the fitted energy-temperature curve and the Metropolis criterion, the acceptance ratio varied between 0.20 and 0.25. The elastic network potential in Martini was applied to the individual Bax and cytochrome C molecules, and the Lincs method was used to add the extra potentials on the PO4 atoms of the lipids, to avoid the structural disruptions of the lipid bilayer at high temperature. Long-range electrostatics interactions were controlled by the reaction field algorithm. Short-rang interactions were described by cut-off methods. The temperatures and pressures for the individual system were controlled by V-rescale and Parrinello-Rahman methods with the coupling constants of 1.0 and 12.0 ps. Leapfrog integrator was used to generate the time step of 30 fs in the simulations. For each replica, the simulation time was 3,000 ns (3.0 us). All the analysis in this paper was conducted based on one trajectory at 310 K (body temperature). Thus, the simulation results in this paper should hold the physiological meaning.

### All-atom Simulation Protocols

The initial conformation of the system was obtained from the coarse-grained replica exchange simulations, which had typical residue-residue interactions between cytochrome C and the inner cavity of the pores (0 Ǻ < xy < 10 Ǻ and −5 Ǻ < z < 5 Ǻ). The coarse-grained protein structure was back-mapped into the all-atom coordinates using in-house codes. To reduce the computational load, α6–8 (residue 130–165) in the coarse-grained model were not considered, since they were far away and had no contacts with cytochrome C molecules. We first measured and recorded the positions and orientations for all the helix segments of coarse-grained pores. Then, the all-atom helix structures of the Bax monomer (1F16)^3^ were taken as the references for the superimpositions into the corresponding coarse-grained geometry. Constraining the helix segments, minimization of 50,000 steps and a short 2 ns simulation with 1 fs time step were conducted to relax the loops that connect the helices. The all-atom conformation of cytochrome C (3ZCF)^81^ was mapped to the coarse-grained cytochrome C model by superimposing the backbone atoms. The CHARMM-GUI webserver^82^ was used to establish the all-atom DSPC lipid bilayer that embeds the Bax oligomeric pores. TIP3 water molecules were used to solvate the system. NaCl and CaCl2 with 0.035 M concentrations were employed to mimic the 0.15 M ion strength in solution.

All-atom simulations were performed by the NAMD package using the CHARMM 36 force field with CMAP correction^83^. The NPT simulations were run at the temperature of 310 K and pressure of 1 atm. The RATTLE algorithm was used to constrain the covalent bonds involving hydrogen atoms. Short-range van der Waals (VdW) interactions were calculated by the switch function with the twin-range cutoff at 12 and 14 Ǻ. Long-rang electrostatic interactions were calculated using the force-shifted methods with the cutoff of 14 Ǻ. The velocity verlet integration was used to generate the time step of 2 fs. Simulations were conducted for 40 ns.

All the analysis was performed using the tools in CHARMM, VMD and in-house TCL codes.
